# High-sensitivity ethanol vapor detection using In_2_O_3_@ZnO core–shell nanomeshes fabricated *via* block copolymer templating

**DOI:** 10.1039/d5ra07053h

**Published:** 2026-01-21

**Authors:** Przemyslaw Pula, Zofia Z. Zawistowska, Julia Krol, Magdalena M. Majewska, Maciej Krajewski, Paulina Chyzy, Mariya V. Dobrotvorska, Maria Kaminska, Mikołaj Lewandowski, Pawel W. Majewski

**Affiliations:** a Faculty of Chemistry, University of Warsaw Warsaw 02093 Poland pmajewski@chem.uw.edu.pl; b Faculty of Physics, University of Warsaw Warsaw 02093 Poland; c NanoBioMedical Centre, Adam Mickiewicz University Poznań 61614 Poland lewandowski@amu.edu.pl

## Abstract

Metal-oxide semiconductor nanowires are promising building blocks for high-performance gas sensors due to their high specific surface area and tunable electronic properties. In this work, we adapted a single-step synthesis based on block copolymer templates to fabricate indium oxide (In_2_O_3_) nanowires subsequently coated with a thin layer of zinc oxide (ZnO) *via* atomic layer deposition (ALD). The optimized core–shell heteronanostructures, featuring a 10 nm-thick ZnO shell and annealed at 400 °C, exhibited a markedly enhanced electrical response measured as a resistance ratio in the absence and presence of ethanol vapors (*R*_0_/*R* ≈ 245 at 100 ppm), as well as high sensitivity (≈2.28 ppm^−1^) in the 10–100 ppm range as compared to bare In_2_O_3_ nanowires (response ≈ 120, sensitivity ≈ 1.01 ppm^−1^). This increase in response and sensitivity is related to the electronic structure of the In_2_O_3_@ZnO heterostructure. Additionally, the core–shell configuration shows promising long-term stability, maintaining high response performance in both dry and ambient humidity conditions. The structural characterization revealed a highly porous and interconnected nanowire architecture of the sensing material and showed that high-temperature annealing significantly improves the crystallinity of both the In_2_O_3_ core and the ZnO shell. The combination of high sensitivity and robust response underscores the potential of these porous core–shell heteronanostructures with a high surface-to-volume ratio for low-concentration detection of ethanol and potentially also other volatile organic compounds, offering a promising avenue for advanced gas-sensing applications.

## Abstract 2

Metal-oxide semiconductor nanowires are promising building blocks for high-performance gas sensors due to their high specific surface area and tunable electronic properties.

## Graphical abstract

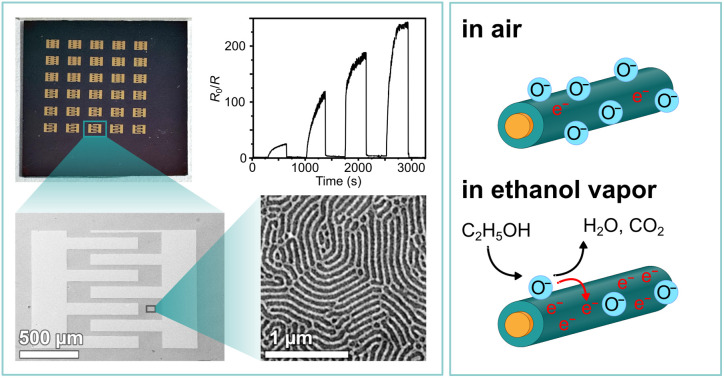

## 1. Introduction

Metal oxide semiconductors (MOS) have garnered significant attention in scientific and industrial fields due to their tunable electrical, optical, and mechanical properties.^1^ These attributes can be significantly altered through surface functionalization or doping, making MOS materials highly versatile for applications including electronics (*e.g.*, MOSFETs),^2^ energy storage (batteries, supercapacitors),^3,4^ solar cells,^5,6^ catalysis,^7^ and gas sensing.^8,9^ Their low cost, ease of synthesis, processing, and potential for surface modifications further position MOS as attractive candidates for sustainable technological innovations.^10^ Among the diverse applications, MOS-based gas sensors are of particular interest, spurred by growing concerns about air quality and associated health risks. Volatile organic compounds (VOCs), emitted by everyday materials, such as furniture, flooring, and electronic devices, pose a significant threat to indoor air quality and underscore the need for reliable real-time pollutant monitoring.^11^

MOS-based gas sensors have traditionally been fabricated from bulk crystalline materials by grinding^12^ or precipitation,^13^ followed by shaping into thin pellets. Although bulk MOS exhibit promising characteristics for chemiresistive gas sensing, *i.e.*, mechanical durability, chemical stability, and low production cost, they often suffer from high power consumption, slow response times, and limited sensitivity at room temperature.^14^ These drawbacks can be mitigated by transitioning to MOS nanostructures, which leverage nanoscale dimensions to achieve enhanced gas-sensing capabilities.^15^ Recent advancements in nanostructured materials, particularly one-dimensional (1D) nanowires (NWs), have significantly improved the performance of MOS-based sensors.^16^ Nanowires offer an exceptionally large specific surface area, improving gas adsorption and response times relative to bulk materials.^17^ Their high surface-to-volume ratio also makes them attractive for catalytic applications.^18^ Various techniques, including chemical vapor deposition (CVD),^19^ hydrothermal or solvothermal methods^20^ as well as vapor–liquid–solid (VLS) deposition,^21^ have been developed to synthesize MOS nanowires. However, these methods can be expensive, require specialized processing and deposition equipment, and do not provide precise control over the composition and morphology of synthesized nanomaterials.

An alternative route involves using self-assembling block copolymers (BCPs) as precise morphological templates to guide the formation of MOS nanostructures. BCPs, composed of chemically distinct covalently linked polymer chains, spontaneously organize into well-defined nanoscale morphologies, such as lamellae, cylinders, or gyroids.^22^ Selective infiltration of these morphologies with metal precursors, followed by removal of the polymer matrix (through oxygen plasma treatment or thermal ashing), produces highly ordered MOS nanostructures.^23–26^ This bottom-up strategy offers a scalable, cost-effective, and versatile approach to creating MOS nanostructures with tunable morphologies.

In the field of gas sensing, transition metal oxides, such as SnO_2_, ZnO, and Fe_2_O_3_, have been extensively studied due to their intrinsic properties and proven efficacy in detecting various gases, including VOCs.^15,27–29^ Similarly, main group metal oxides, such as indium oxide (In_2_O_3_), have demonstrated significant potential for gas detection.^30–32^ In this context, numerous studies have explored the use of BCP templates for the synthesis of MOS inorganic replicas,^33–39^ including In_2_O_3_.^40^ Indium oxide is an n-type semiconductor that is optically transparent in the visible range, being commonly used in thin film or nanostructured forms as a transparent conductive electrode in solar cells and optoelectronic devices.^41–43^ Its high surface activity and doping versatility make it well suited for detecting both oxidizing and reducing gases.^44^ Nanostructures composed of this material have already demonstrated their sensing capabilities for various gaseous inorganic compounds, including CO,^45^ NH_3_,^45^ NO_2_,^46^ and VOCs, such as acetone, ethanol, or toluene.^45,47^ The performance of MOS-based sensors can be enhanced by combining it with a second metal oxide in the form of a heterostructured material. While In_2_O_3_-based mesoporous heterostructures have been reported,^48–50^ a particularly effective design is the core–shell nanowire geometry, in which a core MOS is coated with a different material. The core–shell approach has been successfully applied to various binary systems, as comprehensively reviewed by Long *et al.*^51^ The heterojunction formed at the core–shell interface creates electronic depletion layers, enhancing signal amplification and selectivity. This approach has been successfully demonstrated for In_2_O_3_-containing composites, allowing the detection of single-ppm concentrations of VOCs,^52,53^ triethylamine,^54^ and *n*-butanol.^55^

Here, we describe the fabrication and characterization of In_2_O_3_-core–ZnO-shell (In_2_O_3_@ZnO) nanowire-based chemiresistive sensors applicable for the detection of ethanol vapor, which can be considered a model VOC. Our scalable, one-step process utilizes a self-assembling BCP polystyrene-*block*-poly(2-vinylpyridine) (PS-*b*-P2VP) that natively forms cylindrical morphologies of P2VP embedded in PS.^56^ The P2VP domains are selectively infiltrated with an indium precursor (indium acetylacetonate) during co-deposition of the BCP and acetylacetonate mixture by spin-casting onto a solid substrate. Subsequently, the BCP template is removed by plasma ashing to form the In_2_O_3_ replica. These nanowires are then conformally coated with ZnO layers of varying thicknesses, using atomic layer deposition to achieve a well-defined In_2_O_3_@ZnO core–shell architecture. The resulting heterostructured NWs exhibit superior ethanol sensitivity compared to uncoated In_2_O_3_ NWs. The results highlight this facile strategy's potential for producing high-performance gas sensors in response to the growing demands for reliable, real-time monitoring of low concentrations of organic air pollutants.

## 2. Experimental

### 2.1. Materials

Cylinder-forming polystyrene-*block*-poly(2-vinylpyridine) (PS-*b*-P2VP) BCP with the composition 182 kg mol^−1^-*b*-77.0 kg mol^−1^ (PDI = 1.03), later abbreviated as C259, was purchased from Polymer Source. Anhydrous acetylacetonate salt of indium(iii) (In(acac)_3_) (97%) was purchased from Sigma-Aldrich Inc. and dissolved in GPC-grade toluene (Carl Roth) mixture with 3,4,5-trimethoxytoluene (97%, Sigma-Aldrich, TMOT) to yield a 3% w/w stock solution.

### 2.2. Thin film casting

Standard single-sided polished 15 mm × 15 mm electronic grade B-doped Si wafers (∼500 µm thick) with a 〈100〉 crystallographic orientation and a native ∼2 nm SiO_2_ layer were used as substrates. The wafers were purchased from ITME, Poland. C259 BCP was dissolved in dry toluene with the molecular sieve-dried cosolvent TMOT. C259 was mixed with In(acac)_3_ stock solution in the appropriate metal to vinylpyridine molar ratio (*e.g.*, 1 : 4, 3 : 8, 1 : 2 and 1 : 1) to yield the final polymer concentration of 1.5%. The solutions were filtered with a 0.2 µm PTFE syringe filter prior to use. The silicon substrates were cleaned with oxygen plasma (PE-25, Plasma Etch, 150 mTorr O_2_, 100 W RF power, 180 s) immediately before spin-coating the mixture at room temperature for 120 s (SPIN150i, SPS-Europe). Spin-coating rate adjustment (2000–6000 rpm) was used to control the final thickness of the dried films, as verified by white light spectral reflectance (WLSR) (Filmetrics F-20 UV, KLA Instruments).

### 2.3. Plasma ashing

Plasma oxygen etching (150 mTorr O_2_, 100 W RF power, 600 s) was used to oxidize and remove the polymer matrix and to decompose the acetylacetonate metal precursor sequestered into the 2VP block yielding the In_2_O_3_ replica of BCP morphology.

### 2.4. In_2_O_3_ sensing layer fabrication

A ∼1 µm-thick silicon dioxide insulation layer was thermally grown on a silicon substrate (1150 °C, Czylok FCF5). To facilitate rapid fabrication of sensors with 4 or 6 NW layers, In(acac)_3_-infused C259 bilayers (∼100 nm thick films) were deposited in two or three consecutive spin-coating steps, respectively. Each step used a 1.5% C259 solution in 10% TMOT–toluene with an In : VP ratio of 3 : 8 and a spin-coating duration of 120 seconds. After this procedure, the sample was placed on a hot plate maintained at 60 °C to accelerate the evaporation of TMOT. The sample was then subjected to oxygen plasma ashing. This sequence was repeated two or three times to achieve the desired thickness of the sensory layer. Rapid thermal annealing (RTA) was performed at 400 °C in an O_2_ atmosphere for 5 minutes in a custom-built setup with a 70 W IR laser diode source to improve the degree of crystallinity and to enhance the electrical conductivity of the fabricated mesh.

### 2.5. High-temperature thermal gradient annealing

A custom-designed aluminum temperature gradient device *i.e.*, “diving board” was used to support a 1 cm × 4 cm silicon substrate in a suspended configuration, maintaining a single-point thermal contact. The holder was placed inside a thermostated vacuum chamber (≈200 mTorr, 200 °C) equipped with a transparent quartz window. A thermal gradient was generated using a 30–70 W 980 nm laser shaped into a line focus directed at the distal end of the substrate. The resulting temperature profiles across the substrate surface were verified using a thermal imaging camera (Optris, Xi400). A four-layer In_2_O_3_ NW nanomesh coated on a Si substrate was plasma ashed in oxygen and subsequently annealed at 200–470 °C for 5 min in vacuum.

### 2.6. Electrodes' deposition

Eight interdigitated finger-shaped electrodes (600 µm long and 100 µm wide, with a 40 µm channel width; and total channel length = 2.8 mm) were patterned with a positive photoresist (MaP-1215, Micro Resist Technology GmbH), subsequently washed with a developer (MF-CD26, Micro Resist Technology GmbH), and illuminated using Polos MicroPrinter maskless system featuring a 435 nm writing wavelength and a stepper ensuring seamless multi-frame exposures of the substrate. After thermal evaporation (nanoPVD-T15A, Moorfield) of 4 nm of Cr (interface layer) followed by 75 nm of Au (electrode material), the undeveloped resist was removed by the lift-off process using 1-methyl-2-pyrrolidone (99.5%, anhydrous, Sigma-Aldrich) at 60 °C. Finally, the organic residues were removed by immersion in acetone with mild sonic agitation and rinsing with ultrapure water.

### 2.7. Shell fabrication

ZnO thin films were deposited using a commercial ALD system (Beneq TFS-200) operated in thermal mode. The depositions were conducted on three types of substrates: (i) silicon wafers with a native oxide layer (∼2 nm) for thickness calibration, (ii) silicon substrates with ∼1 µm of thermally grown SiO_2_, and (iii) multilayered, interconnected In_2_O_3_ nanowire structures intended for gas sensing applications. A zinc precursor (diethylzinc, DEZn, 99.9%) and an oxygen precursor (water, 99.99%) were purchased from Sigma-Aldrich and kept at room temperature. The inert gas flowing through the system was nitrogen with 5 N purity. All depositions were performed at a chamber temperature of 200 °C. One cycle of a ZnO deposition process consisted of a pulse of 120 ms of DEZn followed by 8 s of purging time, then an H_2_O pulse of 120 ms followed by 8 s of purging time. The ZnO growth rate under these process parameters was consistent across consecutive depositions and was determined to be 1.7 Å per cycle verified by white light spectral reflectometry (WLSR; Filmetrics F20-UV) and X-ray reflectivity (XRR; Bruker D8 Discover).

### 2.8. Sensor testing

The sensor's response to ethanol vapor was examined with an electrical probe station with a hermetic test chamber (HCP622G-PS, Instec Inc.), equipped with a heating station and a source measure unit (Keithley 2450, Keithley Instruments) operating in a 2-wire configuration with constant voltage bias (10 V).

To generate controlled concentrations of ethanol vapor, a custom-built gas mixing system consisting of two independent gas lines was constructed. The first line directed dry air (Oxford Cryosystems AD51, RH ∼3% at ambient temperature ∼23 °C) through a bubbler filled with anhydrous ethanol (Sigma-Aldrich, ≥99.9%) at a fixed flow rate of 2 dm^3^ min^−1^ to produce ethanol-enriched air. The second line supplied dry air alone at a constant flow rate of 7 dm^3^ min^−1^. The flow of ethanol-containing air was regulated using a mass flow controller (MFC) operating in the 10–500 sccm range, allowing precise adjustment of its dilution with the dry air stream. The two streams were combined in a dedicated mixing compartment, and the resulting gas mixture was delivered to the test chamber (volume ∼600 cm^3^) after initial equilibration. A three-way valve controlled the direction of flow – either allowing the mixed gas to enter the chamber during the exposure phase or switching to a pure dry air stream for purging. This setup enabled precise control over the ethanol vapor concentration introduced into the test environment.

For the experiments testing cross-sensitivity of the sensor to water vapor, the air stream carrying ethanol vapors was humidified to about 12 000 ppm H_2_O (≈40% relative humidity, %RH at 25 °C). The exact humidity level was recorded by the commercial RH sensor (SGP-40, Sensirion AG) located in the immediate vicinity, up-stream from the test chamber.

To ensure the accuracy and stability of the ethanol concentration, a reference commercial gas sensor (SGP-40, Sensirion AG), dedicated for ethanol detection in the range of 0.5–1000 ppm, was placed in-line at the mixing compartment. Along with a VOC sensing, the sensor is equipped with the humidity sensor. The reference sensor continuously monitored the ethanol concentration and enabled fine-tuning of the bubbler line flow rate to achieve the desired value. The SGP-40 sensor was positioned in a dedicated pre-chamber before the hermetic chamber to avoid temperature-related signal value drift. After each exposure cycle, ethanol was purged from the test chamber by isolating the bubbler line and allowing only the dry air stream (flow rate 7 dm^3^ min^−1^) to flow into the chamber until the sensor indicated complete removal of ethanol.

### 2.9. Scanning electron microscopy (SEM)

The morphology of plasma-etched samples was examined under a field emission SEM (Zeiss Merlin) operating at 3 keV and equipped with an in-lens detector of secondary electrons.

### 2.10. Transmission electron microscopy (TEM)

The morphology of the In_2_O_3_ NWs coated with ZnO layers of varying thickness (0, 5, 10, 20, and 30 nm) was examined using transmission electron microscope (JEOL JEM1400, 120 kV) operating at an accelerating voltage of 80 kV. For TEM analysis, a small portion of the plasma-etched In_2_O_3_ NWs, previously subjected to ZnO ALD coating, was gently transferred onto carbon-film-coated TEM grids (400 mesh) by mechanical scraping.

### 2.11. Powder X-ray diffraction (XRD)

The crystallographic structure of the samples was studied by XRD. In(acac)_3_ dissolved in a TMOT : toluene mixture was cast on a background-free silicon substrate (*i.e.* showing no reflexes in the 2*θ* measured range) ((911)-cut, Siltronix). For the core–shell sample fabrication, the 50 nm-thick ZnO coating was deposited *via* ALD at 200 °C (see Section 2.7). The subsequent high-temperature annealing was conducted for 5 min at 400 °C in an O_2_ atmosphere. The diffractograms were collected using a powder X-ray diffractometer (D8 Discover, Bruker Inc.) with collimated Cu K_α_ radiation (0.154 nm) in the 15–60° 2*θ* range in the locked-coupled mode. Data fitting and analysis were performed using Topas software (Bruker Inc.).

### 2.12. X-ray photoelectron spectroscopy (XPS)

The chemical composition of fabricated sensor materials was determined by XPS. The measurements were performed in an ultra-high vacuum (UHV) system with a base pressure of 5 × 10^−10^ mbar. The spectra were acquired using an Al K_α_ (1486.6 eV) X-ray source and a hemispherical electron energy analyzer (Omicron). A pass energy of 50 eV was used for the collection of the survey spectra and 20 eV for the regions. The results were analyzed using CasaXPS computer software (Casa Software Ltd). For the measurements, indium-infused thin films were spin-coated onto silicon substrates coated with a thin gold layer deposited over a chromium wetting layer. The role of gold was to prevent samples from charging during the measurements and serve as an energy calibration reference. Three types of samples were investigated: pure In_2_O_3_ NWs, In_2_O_3_ NWs coated with a 2 nm-thick ZnO layer, and In_2_O_3_ NWs coated with 5 nm of ZnO. The binding energy scale calibration of pure and 2 nm ZnO-coated NWs was made based on the position of the Au 4f_7/2_ peak (84.0 eV) of the substrate. For the 5 nm ZnO-coated sample, the signal from gold was not detectable due to the excessive thickness of the ZnO layer, so the C 1s peak of adventitious hydrocarbons (285.3 eV) was used as a reference. For the fittings, Shirley background subtraction and a Gaussian–Lorentzian lineshape (linear combination in a 50%/50% ratio) were applied.

## 3. Results and discussion

### 3.1. Synthesis and morphological characterization of annealed In_2_O_3_ and In_2_O_3_@ZnO core–shell NW multilayer meshes

In our recent work, we have introduced a straightforward strategy for depositing metal oxide NWs by utilizing a BCP template in combination with a volatile solvent/non-volatile cosolvent mixture that prolongs solvent evaporation, thus promoting more ordered, metal-infused BCP domains.^56^ This method was successfully validated for five different transition metal oxide materials: V_2_O_5_, Mn_2_O_3_, Fe_2_O_3_, and chromium and cobalt oxides. Based on this approach, we have investigated the co-self-assembly of indium(iii) acetylacetonate and cylindrical PS-*b*-P2VP BCP with a P2VP minority block and *M*_w_ = 259 kg mol^−1^ at various In : VP ratios during casting from a 10% 3,4,5-TMOT–toluene solvent mixture. The introduction of a low-volatile TMOT component into the spin-casting mixture prolongs the solvent-drying process and greatly enhances the degree of order in the resulting precursor-infused BCP thin films.

**Fig. 1:**
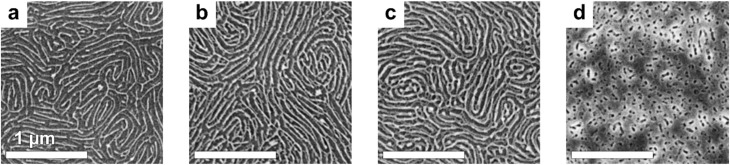
SEM images of oxygen plasma-etched indium oxide nanostructures derived from cylindrical PS-*b*-P2VP (C259) BCP blended with indium(iii) acetylacetonate in different In : VP stoichiometric ratios: (a) 1 : 4, (b) 3 : 8, (c) 1 : 2, and (d) 1 : 1. All samples were spin-casted at the same speed of 2000 rpm from a 1.5% BCP/10% TMOT–toluene solution at room temperature for 120 s, followed by evaporation at 60 °C.

Fig. 1 demonstrates the efficiency of this approach in obtaining In_2_O_3_ nanowire replicas of C259. At an In : VP stoichiometric ratio of 1 : 4 (Fig. 1a), the morphology comprises of two overlapping layers of relatively thin (∼24 nm) horizontal cylinders resulting from the collapse of the bilayer-thick BCP film during plasma ashing.^57,58^ Increasing the ratio to 3 : 8 (Fig. 1b) leads to a higher inorganic material content, resulting in nanowires that appear more continuous and better defined over larger distances in the as-cast replica. Further increasing the In : VP ratio to 1 : 2 (Fig. 1c) begins to interfere with the self-assembly process, as evidenced by a greater number of point defects, indicating that excess metal precursor partially disrupts the BCP microphase separation process. At the highest tested ratio of 1 : 1 (Fig. 1d), significant aggregation of the inorganic component is observed, which compromises the selective coordination between the indium precursor and the P2VP blocks, ultimately leading to the loss of long-range order. Based on these observations, the In : VP ratio of 3 : 8 was selected as the optimal platform for the synthesis of In_2_O_3_ nanowires.

**Fig. 2:**
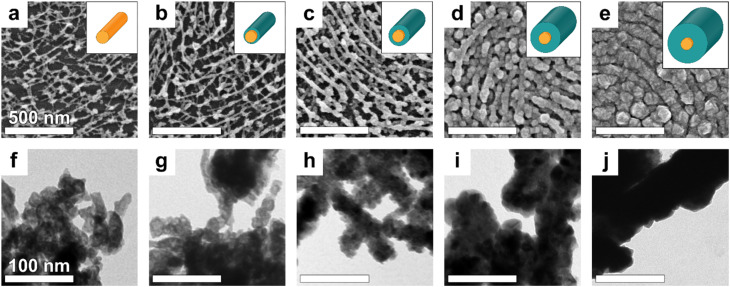
(a–e) SEM and TEM (f–j) morphologies of 4-layer In_2_O_3_ NW films cast on a silicon wafer from PS-*b*-P2VP C259 In : VP 3 : 8 10% TMOT solution in toluene: (a and f) without ZnO, and with ZnO ALD-deposited thin films of (b and g) 5 nm, (c and h) 10 nm, (d and i) 20 nm, and (e and j) 30 nm thickness. The images were acquired after the high-temperature treatment. In the insets of the top row, the corresponding graphical illustrations depict the NW's orange core and teal shell while preserving their actual size proportions. Top row SEM images (a–e) scale bar is 500 nm; bottom row TEM images (f–j) scale bar is 100 nm.

The effectiveness of a gas sensor depends greatly on the specific surface area of the material since a more developed surface area provides more sites where the target gas molecules can adsorb. The C259 PS-*b*-P2VP BCP was chosen for its characteristic cylinder center-to-center spacing (*L* ≈ 70 nm) and the In_2_O_3_ NW diameter (*d* = 24 nm). This geometry yields a high specific surface area of ∼23 m^2^ g^−1^ (estimated based on geometric considerations). Such an architecture provides sufficient space for the deposition of a ZnO shell without forming an overcoated “crust” that would hinder diffusion and reaction in the active layer, lowering the materials' surface-to-volume ratio. The tested single-layer NW sensors displayed large (≈1 GOhm) resistance due to the low surface coverage of the substrate (Fig. S1), which would result in limited current-flow percolation between the contact electrodes. To address these limitations and mitigate issues associated with low-current measurements in sensing experiments, we deposited four layers of NWs.^59^ As shown in Fig. 2a and f, the resulting nanomesh morphology is relatively sparse, and the bare In_2_O_3_ NWs do not obscure the substrate below.

To form the shell layer, we have used ALD to coat the 4-layer In_2_O_3_ NW films with a conformal ZnO layers of 5 nm to 30 nm thickness by adjusting the number of diethyl zinc and water deposition cycles using a calibrated ALD recipe. SEM images presented in Fig. 2b–d confirm that for 5 nm, 10 nm and 20 nm ZnO thickness the structure remains porous overall; however, as the ZnO thickness increases, the NWs appear more densely packed, and the internal void volume decreases (Fig. S2). This densification could hinder deeper penetration of sensed molecules and trap them in the nanostructure, making diffusion and desorption more difficult. Ultimately, a thicker 30 nm ZnO coating leads to a morphological transition to a continuous film (Fig. 2e), which reduces the overall surface-to-volume ratio and adversely affects gas sensing performance.^60^

Further morphological analysis confirming the core–shell architecture was carried out using transmission electron microscopy (TEM). Examination of the sensory material scrapped off the silicon substrates and transferred onto TEM grids revealed that the In_2_O_3_ core, visible as the darker contrast region, possesses a diameter of approximately ∼25 nm (Fig. 2f). Subsequent TEM images clearly show the progressive increase in ZnO shell thickness, consistent with the targeted thickness in accord with the employed ALD deposition protocol (Fig. 2g–j).

**Fig. 3:**
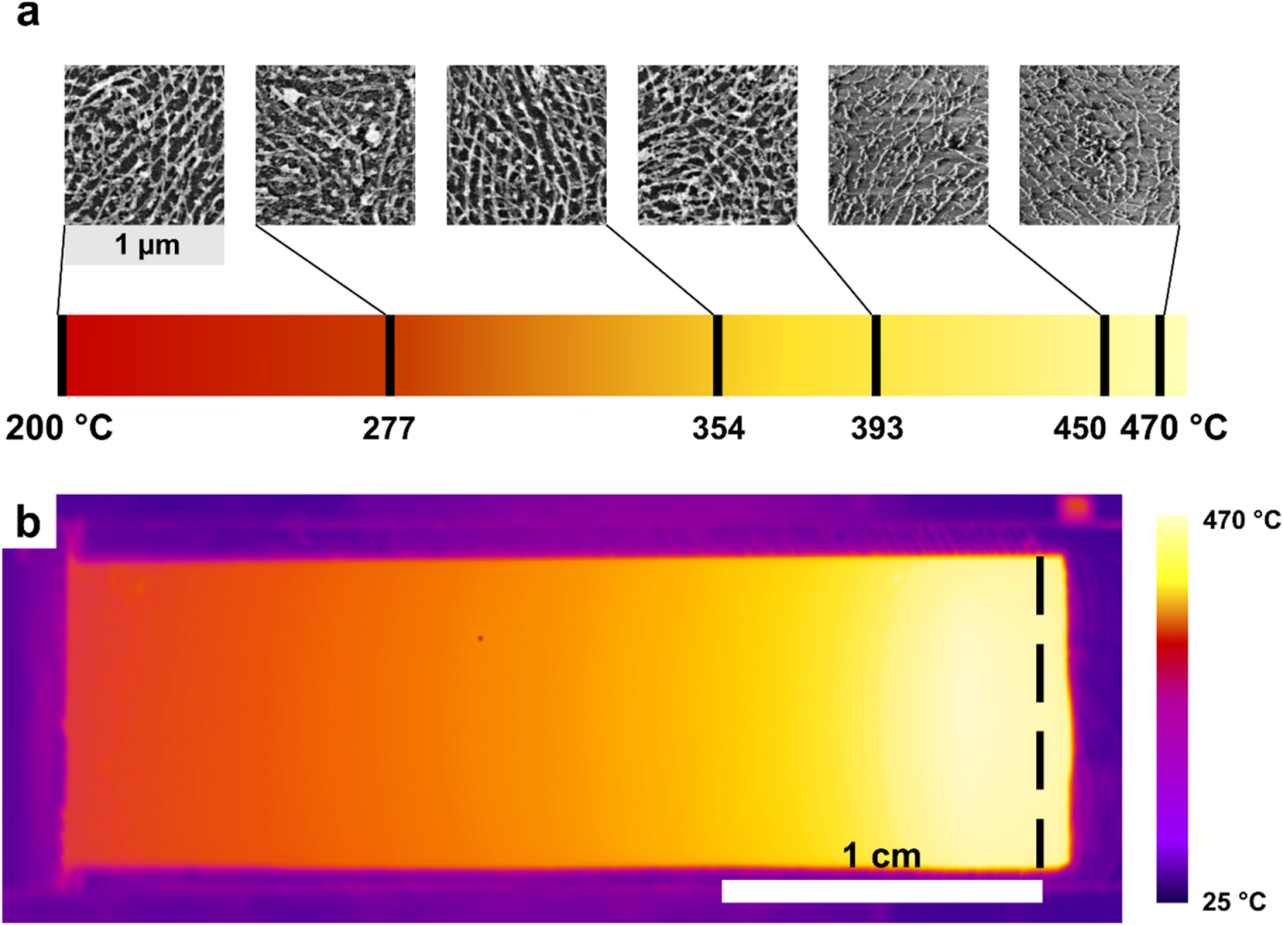
(a) SEM images of 4-layer In_2_O_3_ nanowire films cast on a silicon wafer from PS-*b*-P2VP C259 In : VP 3 : 8 10% TMOT solution in toluene acquired at different locations across the substrate, corresponding to increasing local temperatures along the thermal gradient. The specific temperature at each position is indicated above the respective image. Each image covers an area of 1 µm × 1 µm. (b) Thermal profile captured using an IR thermographic camera. The dashed line marks the incidence of the line-shaped laser beam.

To maximize the In_2_O_3_ NWs' signal output for subsequent ethanol sensing experiments, we have applied post-annealing treatments at various temperatures to enhance both the crystallinity and conductivity of the material.^61^ To perform the single combinatorial experiment, a rectangular silicon substrate was coated with four layers of In_2_O_3_ nanowires and subjected to plasma ashing prior to high-temperature annealing. A photograph of the custom-built aluminum gradient-annealing setup used in this procedure is provided in the SI (Fig. S3). The annealing was carried out in a thermostated chamber kept under low-pressure conditions (∼200 mTorr) in air to ensure the formation of a linear thermal gradient across the substrate. The gradient was established using a line-shaped infrared laser (30–70 W), producing a temperature difference (Δ*T*) of approximately 270 °C. Based on the SEM images collected (Fig. 3a), annealing temperatures exceeding 400 °C for 5 minutes result in physical damage to the In_2_O_3_ NWs deposited on the Si/SiO_2_ substrate. Consequently, to avoid possible damage to the NW continuity, we have selected 400 °C as the annealing temperature.

### 3.2. Crystallographic and chemical characterization of native In_2_O_3_ and core–shell In_2_O_3_@ZnO NWs

**Fig. 4:**
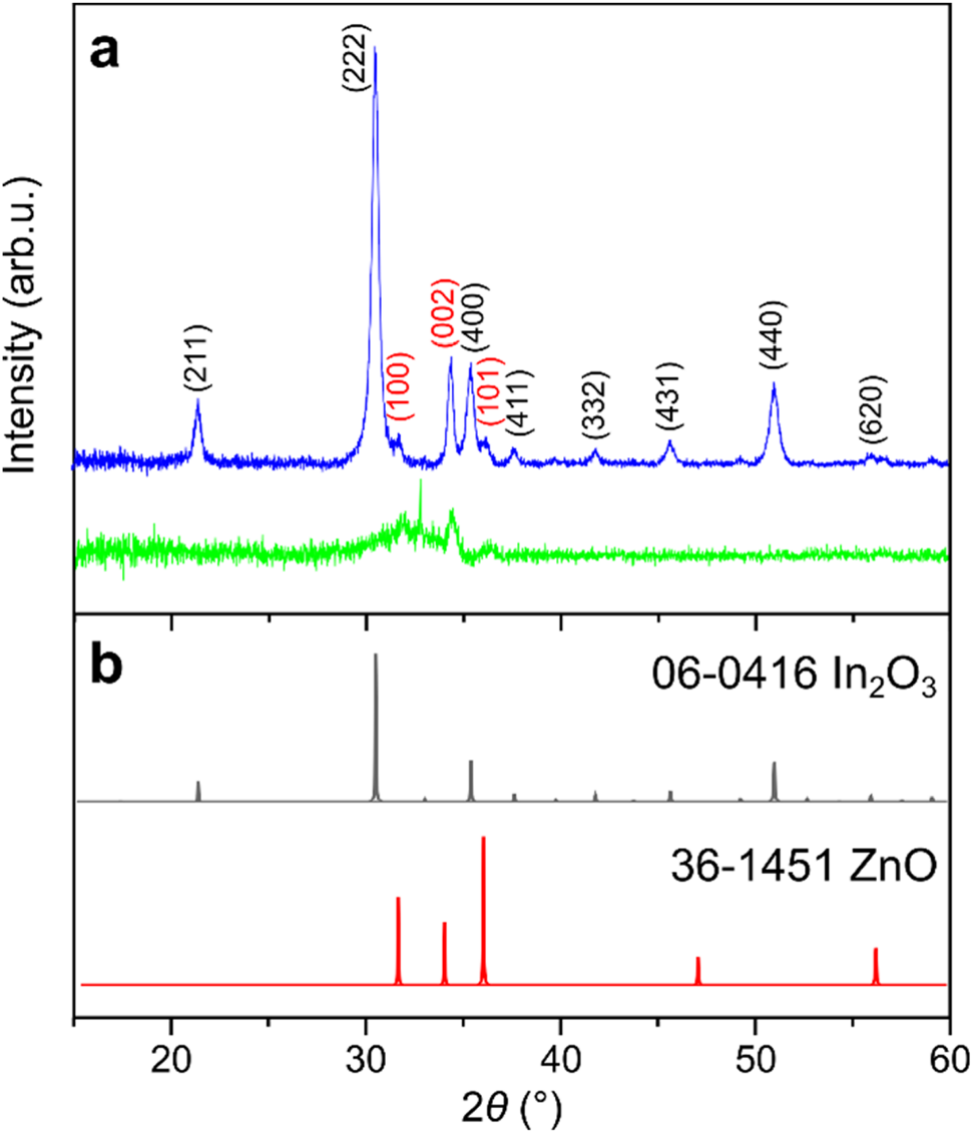
(a) XRD patterns of In_2_O_3_ C259 replicas with ALD-deposited 50 nm ZnO shell, recorded before (green curve) and after rapid thermal annealing in O_2_ at 400 °C for 5 minutes (blue curve). The *hkl* crystallographic planes are marked for the respective reflexes (In_2_O_3_ – gray, ZnO – red). (b) Simulated diffraction patterns of In_2_O_3_ (gray curve) and ZnO (red curve), derived from their respective CIF entries, alongside the corresponding JCPDS card numbers.

To determine the crystallographic structure of ZnO-coated In_2_O_3_ NWs derived from the C259 template, we used XRD. Prior to thermal annealing, bare indium oxide NWs were amorphous, as indicated by the absence of reflexes in diffractograms (Fig. S4). Upon depositing a 50 nm-thick ZnO coating in the ALD reactor at 200 °C, broad peaks characteristic of the hexagonal wurtzite (*P*6_3_*mc*) ZnO phase appeared (Fig. 4a, green curve), indicating partial crystallinity. A weak and broad reflection from the bixbyite (body-centered cubic, Ia3̄) In_2_O_3_ phase also emerged, suggesting that indium oxide began to transform from amorphous to cubic during the ALD process. Subsequent annealing at 400 °C in oxygen yielded sharp and intense peaks, revealing a high degree of crystallinity in both cubic In_2_O_3_ and the wurtzite ZnO phase (Fig. 4a, blue curve). Comparison with simulated diffraction patterns for In_2_O_3_ and ZnO, derived from CIF entries and corroborated by corresponding JCPDS cards, confirmed these assignments.

Next, we have determined the chemical composition of synthesized nanomeshes using XPS. We have examined uncoated NW replicas and those coated by ALD with 2 nm or 5 nm of ZnO. The core–shell materials analogous to those shown in Fig. 2, were annealed in O_2_ at 400 °C for 5 minutes.

The elemental composition was determined from the survey spectra (Fig. S5) by analyzing the intensity of C 1s, O 1s, Au 4f_7/2_, Si 2s, In 3d_5/2_, Cr 2p_3/2_, N 1s, and Zn 2p_3/2_ photoelectron lines, and by taking their relative sensitivity factors (RSFs) into account. In the case of the 5 nm ZnO-coated sample, the signals corresponding to silicon, gold, and chromium were not detected due to the limited photoelectron escape depth (∼5 nm). In the case of 2 nm ZnO-coated sample, these elements were visible, with the exception of chromium, whose peak was overlapping with the Auger signal of zinc.^62^

**Fig. 5:**
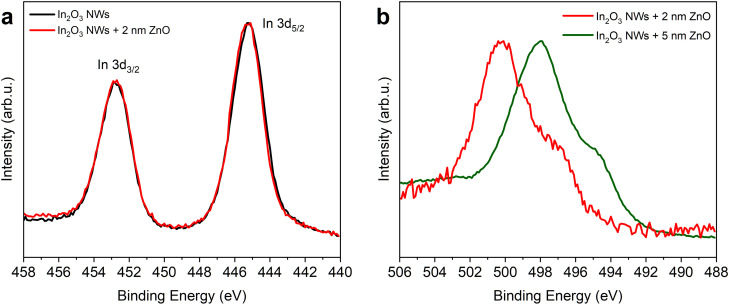
XPS spectra of the In(acac)_3_-infused BCP templates obtained at a 3 : 8 In : VP metal loading ratio, subjected to plasma ashing, and subsequent annealing at 400 °C for 5 min in an O_2_ atmosphere: (a) In 3d core level (uncoated (black line) and coated with 2 nm of ZnO (red)) and (b) Zn LMM Auger spectra (coated with 2 nm (red) and 5 nm of ZnO (green)). The intensities of the spectra were normalized for presentation.

In order to confirm the presence of the In_2_O_3_ phase visible in XRD patterns, we have analyzed the In 3d lines of both the neat indium oxide BCP replica and the ZnO-coated core–shell samples (Fig. 5). Forthe uncoated NWs and the 2 nm ZnO sample, the In 3d_5/2_ peak appeared at 445.1 eV (Fig. 5a, black and red curve, respectively), which is the characteristic binding energy (BE) value of In_2_O_3_ (mean literature value: 444.8 ± 0.6 eV).^62^ The atomic ratio of oxygen (O 1s at 530 eV) to indium (In 3d) was 1.5, further confirming the presence of stoichiometric In_2_O_3_. Notably, ZnO deposition did not alter indium's oxidation state.

Then, we have addressed the structure of the zinc oxide layer by analyzing the recorded Zn 2p signals. Even though the Zn 2p core level shift is generally small relative to the position of metallic zinc and zinc oxide, the position and shape of the Zn Auger LMM line is highly sensitive to the chemical state.^62,63^ In the case of the 5 nm ZnO sample (Fig. 5b, green curve), the position of the Zn LMM peak was 497.9 eV, which is characteristic of ZnO.^62^ In the case of the 2 nm ZnO sample (Fig. 5b, red curve), the Auger peak was shifted to higher binding energy, reflecting lower kinetic energy (KE) of the emitted Auger electrons, more characteristic of zinc hydroxide.^63^ Thus, the obtained results indicate that 2 nm thickness is not sufficient to form a well-ordered ZnO layer, while 5 nm is.

To address the potential calibration inconsistencies and sample charging effects, we have evaluated the Auger parameter (*α̇*), defined as *α̇* = KE(Zn LMM) + BE(Zn 2p_3/2_), where KE is the kinetic energy of the Zn L_3_M_4,5_M_4,5_ Auger line. For the 5 nm ZnO sample, the *α̇* = 2010.1 eV, which is consistent with ZnO. The 2 nm ZnO sample yielded *α̇* = 2009.4 eV – a value associated with Zn(OH)_2_ and more complex compounds, such as Zn_5_(CO_3_)_2_(OH)_6_.^63^ This assignment, supported by the shape of O 1s and C 1s spectra, suggests that zinc hydroxides can form at the surface of thinner ALD ZnO layers on In_2_O_3_. Such hydroxide formation may be linked to higher reactivity of zinc at the ZnO–In_2_O_3_ interface, where a greater number of undercoordinated Zn atoms could favor Zn(OH)_2_-like species over pure ZnO.

In order to determine the molar and weight ZnO-to-In_2_O_3_ ratio, we have first addressed the 2 nm ZnO sample, *i.e.*, the only one where both Zn and In signals could be detected. The analysis required considering the attenuation of signals originating from indium oxide due to the scattering of emitted photoelectrons by the Zn(OH)_2_ overlayer. The intensity attenuation can be estimated using the equation: *I*_i_ = *I*_0i_ exp(−*d*/*λ*_i_), where *I*_i_ is the observed intensity (area) of the attenuated signal, *I*_0i_ is the real intensity, *d* is the thickness of the covering layer and *λ*_i_ is the photoelectron inelastic mean free path (IMFP).

The IMFP depends on the KE of emitted photoelectrons and for inorganic materials lies in the range of 1–5 nm.^64^ The exact value of the IMFP at the kinetic energy corresponding to the specific core level can be calculated using the TTP-2M equation, as implemented in the NIST IMFP database (SRD 71), or can be reasonably approximated as *λ* ∼ KE^0.67^.^65^ To estimate *d*, one can use the intensity ratio (divided by the respective RSFs) of two indium lines lying at different KE values: In 3d_5/2_ (KE = 1041 eV) and In 3p_3/2_ (KE = 819 eV) (see the survey spectra in Fig. S5), through the equation:

1 *I*_3d_/*I*_3p_ = exp(*k* × *d* (KE_3p_^−0.67^ − KE_3d_^−0.67^))

For the 2 nm ZnO sample, the calculated ratio was equal to 1.13. Using the *k* × *d* value obtained from eqn (1), the real intensity of the In 3d_5/2_ line can be determined as: *I*_03d_ = *I*_3d_ (*k* × *d*/KE_3d_^0.67^). This estimation shows that the intensity of the recorded In 3d_5/2_ line is roughly half of the real intensity due to the attenuation by the Zn(OH)_2_ overlayer. Having this information, the corrected In/Zn atomic ratio (based on the atomic concentrations provided in the SI) was found to be 2.5. From this ratio, it was further possible to calculate the respective weight concentrations of In_2_O_3_ and Zn(OH)_2_ in the 2 nm ZnO sample, which were found to be 23 wt% Zn(OH)_2_ and 77 wt% In_2_O_3_. For the 5 nm ZnO sample, the zinc oxide layer has a ZnO stoichiometry (instead of Zn(OH)_2_), and the thickness of the layer is assumed to be 2.5 bigger than in the case of the 2 nm sample. Taking these considerations into account, the estimated ratio for this sample is 35 wt% ZnO and 65 wt% In_2_O_3_.

### 3.3. Ethanol sensing using core–shell In_2_O_3_@ZnO meshed NWs

**Fig. 6:**
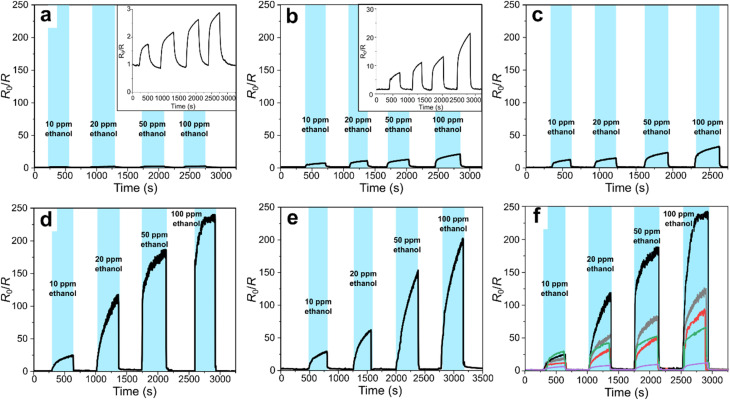
Dynamic responses to ethanol vapor sensor recorded at (a) 250 °C, (b) 300 °C, (c) 350 °C, (d) 400 °C, and (e) 450 °C for a 4-layer thick In_2_O_3_@ZnO NW mesh with Au contact electrodes, fabricated from the C259 template using an In : VP ratio of 3 : 8. The NWs have a 10 nm-thick ZnO shell and were annealed in O_2_ at 400 °C for 5 minutes. (f) Dynamic response characteristics for 4-layer thick In_2_O_3_@ZnO NW sensory meshes with varying ZnO shell thickness. In_2_O_3_ NWs were tested uncoated (gray) and with 5 nm (red), 10 nm (black), 20 nm (green), and 30 nm (purple) ZnO shell. All samples were annealed in O_2_ at 400 °C for 5 minutes prior to testing, sensing measurements were conducted at 400 °C. Each sensor (a–f) was exposed to 10 ppm, 20 ppm, 50 ppm, and 100 ppm of ethanol in 5-minute intervals, followed by a 5-minute purge with pure air.

The optimal operating temperature of the sensor was determined by evaluating the response parameter (Fig. 6) derived from the corresponding raw resistance data (Fig. S6) of a 4-layer In_2_O_3_ NW sample, annealed at 400 °C and coated with a 10 nm ZnO layer, in a range of 250–450 °C, which is a typical operating temperature range for MOS sensors.^66^ The sensor response was defined as *R*_0_/*R*, where *R*_0_ is the electrical baseline resistance in dry air, and *R* is the resistance measured in the presence of ethanol vapor. An SEM micrograph of a representative sensor with patterned gold electrodes used for the measurements is provided in the SI (Fig. S6a).

At 250 °C operating temperature, the core–shell sensor exhibited a gradual and reversible decrease in resistance upon exposure to increasing ethanol concentrations (Fig. 6a). Given the very small resistance change (*R*_0_/*R* < 3), it indicates a relatively low surface reactivity at this temperature (Fig. S6b). At 300 °C, the sensor's baseline resistance increased, and sharper transitions were observed between exposure and recovery phases. We noted an emergence of partial saturation effect at higher ethanol doses (Fig. 6b and S6c). At 350 °C, well-defined resistance drops were noted for each ethanol concentration, with good reproducibility and faster response/recovery dynamics (Fig. 6c and S6d), correlating with the highest response ratio (*R*_0_/*R* ≈ 33 at 100 ppm), confirming enhanced surface reaction adsorption/desorption kinetics with a stable baseline resistance. At 400 °C (Fig. 6d and S6e), the sensor performance was optimal, marked by steep resistance changes, excellent reversibility, and minimal saturation even at 100 ppm, leading to a maximal response (*R*_0_/*R* ≈ 245) and the highest sensitivity of *s* ≈ 2.28 ppm^−1^ (Table S2 and Fig. S7), defined as *s* = (*R*_0_/*R*)/*c*_EtOH_, across the 10−100 ppm range. The slight disadvantage might be a small baseline resistance drift after exposure to higher concentrations. Increasing the temperature to 450 °C resulted in decreased response values (Fig. 6e and S6f) and incomplete recovery at higher ethanol concentrations, likely due to desorption-dominated kinetics and lowered ethanol adsorption efficiency, thereby validating 400 °C as the optimal operating temperature for In_2_O_3_@ZnO (10 nm) core–shell nanowire sensors.

Finally, we have studied the impact of ZnO shell thickness on the ethanol sensing response of In_2_O_3_@ZnO NW meshes (Fig. 6f). The uncoated In_2_O_3_ NWs exhibited a linear increase in response with increasing ethanol concentration (Fig. 6f, gray curve). Adding a 5 nm-thick ZnO shell did not improve the sensor output (Fig. 6f, red curve). In contrast, the highest response was achieved with a 10 nm-thick ZnO coating (Fig. 6f, black curve), which at 100 ppm of ethanol showed a response nearly twice that of the bare In_2_O_3_ NWs (Table S3). The In_2_O_3_ NW sensor coated with a 10 nm ZnO layer displayed a sensitivity of *s* ≈ 2.28 ppm^−1^ (Fig. S8), about an order of magnitude higher than that of neat α-Fe_2_O_3_ NWs reported in our previous work.^56^ Further increasing the ZnO shell thickness beyond 20 nm was reducing the response (Fig. 6f, green and purple curves), in part due to the transition from a one-dimensional NW geometry to a continuous thin film, which diminishes the surface-to-volume ratio.^60^ Interestingly, at the lowest ethanol concentration tested (10 ppm), the highest response was obtained for the 20 nm ZnO shell. This effect can be attributed to the variation of baseline resistance across samples with different ZnO shell thickness (Table S6). A substantially higher baseline resistance (*R*_0_ ≈ 15 MΩ) for the 20 nm ZnO-coated sensor compared to the 10 nm coating (*R*_0_ ≈ 1.3 MΩ, Table S6) likely arises from the increased defect density and enhanced charge carrier depletion at the In_2_O_3_/ZnO heterointerface, which amplify the relative resistance change at low concentration of ethanol (non-saturating available adsorption sites). At higher ethanol concentrations (>20 ppm), however, the thicker ZnO shells likely restrict efficient diffusion of analyte molecules into the nanomesh interior, resulting in preferential saturation of surface-accessible sites and limited interaction with deeper adsorption sites, thereby reducing the overall response. In the case of the thickest ZnO shell (>30 nm), the ALD-grown ZnO layer primarily forms on the top surface of the mesh instead of conformally coating the nanowires. This significantly influences the sensory characteristic of the heterostructure, making it more similar to that of a flat ZnO (see Fig. S9).

A flat ALD-grown ZnO film (Fig. S9), as well as a 6-layer NW mesh (Fig. S10), exhibits the maximum response at 20 nm ZnO layer thickness. However, the response of the 6-layer mesh sensor reached only about 65% of the maximum observed for the 4-layer mesh with a 10 nm coating (Fig. S10). For the 20 nm ZnO coating the 6-layer mesh also showed a lower peak sensitivity (*s* ≈ 1.79 ppm^−1^) compared to 4-layer sensor with a 10 nm coating (Table S4 and Fig. S11). For all the concentrations tested, the sensor's response did not reach a plateau (*i.e.*, it did not fully saturate) within the five-minute exposure period. This incomplete saturation may be due to the limited exchange of ethanol-infused air in the test chamber or to the relatively slow kinetics of adsorption and chemical reactions at the sensor interface.

The enhancement of sensory response at the optimal ZnO thickness likely arises from an optimized space-charge depletion region that modulates the electronic properties at the In_2_O_3_–ZnO interface. Such modulations are strongly linked to the Debye length (*λ*_D_), which represents the distance over which charge carriers screen electric fields in a semiconductor.^67^ When the Debye length is shorter or comparable to the ZnO shell thickness, the charge distribution is altered in a way that enhances carrier interactions at the heterojunction.^68,69^ The estimated Debye length of ZnO ranges from approximately 22 to 35 nm at ∼300 °C.^70,71^ At the operating temperature of 400 °C, the Debye length is expected to increase by approximately 8%.

In the absence of a reductive gas, such as ethanol, oxygen species adsorbed onto the nanowire surface capture free electrons, leading to an increase in resistance. Upon ethanol exposure, surface-bound oxygen reacts with ethanol molecules, injecting free electrons into the nanowire and reducing the electrical resistance. In our multimesh core–shell nanostructures with varying thicknesses (4 and 6 layers), we observed optimal sensor response for the ZnO shell thicknesses of 10 nm and 20 nm, respectively. To understand the discrepancy between the “optimal” ZnO shell thicknesses observed for the 4- and 6-layer nanowire meshes, we examined the evolution of the baseline resistances. For both series, *R*_0_ initially decreases with the addition of thin ZnO coatings, but this trend reverses beyond ∼10 nm, reaching a maximum at ∼20 nm before slightly decreasing again at 30 nm (Table S6 and Fig. S12). SEM imaging (Fig. 2b–e) shows that ZnO begins to coalesce into a more continuous surface layer at thicknesses ≥20 nm in the 4-layer mesh, with this effect even more pronounced in the denser 6-layer architecture (Fig. S2e–h). Because neat ZnO thin films exhibit their highest sensing response at 20 nm thickness (Fig. S9), it is plausible that in the more compact 6-layer mesh the ZnO shell grown on the surface of the mesh increasingly dominates the conduction pathway, diminishing the influence of the In_2_O_3_ core and effectively suppressing the core–shell sensing mechanism. In contrast, the more open 4-layer nanomesh accommodates a thinner, conformal 10 nm ZnO layer that optimally enhances charge transfer at the In_2_O_3_–ZnO interface without inducing significant film coalescence. Also, once the ZnO coating exceeds a certain thickness, coalescence of adjacent wires reduces accessible surface area and hinders gas diffusion into the nanomesh interior, thereby lowering the overall sensory response.

The presence of a ZnO shell on the In_2_O_3_ core nanowires leads to the formation of a type-II heterojunction, as the respective bandgap levels overlap.^72^ Due to its higher work function compared to In_2_O_3_,^73^ ZnO more effectively captures the released electrons (it is more prone to accept electrons to lower its work function). Even though the formation of depletion layers at the In_2_O_3_–ZnO and the inter-wire interfaces introduces potential energy barriers that hinder electron transport,^74^ upon exposure to ethanol, these energy barriers decrease, facilitating charge carrier movement by the electron extraction not only in the shell, but also partially in the In_2_O_3_ core. This results in a preferential electron transport pathway through the In_2_O_3_ core, reducing the number of interfacial crossings and improving overall conductivity. The combined effect of the optimum ZnO shell thickness and the modulation of potential barriers along the percolation path upon gas exposure is the most-likely reason of the enhanced ethanol sensing behavior.^74^

**Fig. 7:**
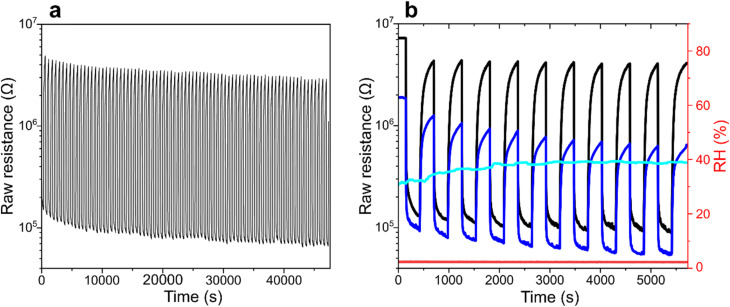
Dynamic raw resistance characteristics of the 4-layer In_2_O_3_@ZnO nanowire mesh sensor with a 10 nm ZnO shell and Au contact electrodes, fabricated from the C259 template at an In : VP ratio of 3 : 8. The nanowires were annealed in O_2_ at 400 °C for 5 min, and sensing measurements were conducted at 400 °C. (a) Raw resistance curve over 80 consecutive cycles of exposure to 20 ppm ethanol in dry air (≈3% RH). (b) Raw resistance curve of the same sensor gathered during 10 cycles of exposure to 20 ppm ethanol in dry air (≈3% RH, black curve) and moisture-enriched air (≈40% RH, blue curve). Each cycle comprised a 5-min exposure followed by a 5-min purge. Real-time RH levels recorded during exposure to dry air and moisture-enriched air are shown by the red and cyan curves, respectively.

We note that the fabricated highly-porous, multilayered bare In_2_O_3_ NWs, as well as those coated with a ZnO shell, exhibit superior signal-response performance compared to many previously reported studies on similar systems (Table 1).^61,74,77,80^ The response (≈120) of our uncoated 4-layer In_2_O_3_ NW sensor (Fig. 7, gray curve) significantly exceeds the values reported for nanowire-based neat In_2_O_3_ ethanol sensors. For instance, Singh *et al.* observed a maximum response of ≈7 for 100 ppm of ethanol at the same operating temperature,^74^ while for lower operating temperatures (90–300 °C) the responses to 100 ppm of ethanol did not exceed value 14.^61,77,79,80^ In the SI (Table S5), we have also included a comparative overview of the surface characteristics of MOS-based commercial sensors available on the market, as reported by manufacturers. Notably, our sensor's sensitivity to ethanol vapor in the 10–100 ppm range offers a ten-fold improvement over the most commonly implemented commercial materials.

**Table 1:** Comparison of ethanol sensing for neat In_2_O_3_ and In_2_O_3_@ZnO nanostructures

Sensing material	Operating temperature [°C]	Concentration [ppm]	*R*_0_/*R* response
In_2_O_3_ thin film^75^	250	300	3.3
In_2_O_3_ nanorods^76^	90	100	7.8
In_2_O_3_ nanofibers^77^	225	100	18.67
In_2_O_3_ nanowires^78^	300	5	2
In_2_O_3_ nanowires^79^	300	100	13.97
In_2_O_3_ nanowires^74^	400	100	7
In_2_O_3_ nanowires^80^	300	200	7
In_2_O_3_ nanowires^61^	300	100	14
In_2_O_3_ nanowires (this work)	400	100	120
In_2_O_3_ : Zn-doped carambola-like nanostructure^81^	220	50	203
ZnO@In_2_O_3_ core–shell nanofibers^77^	225	100	31.87
In_2_O_3_@ZnO core–shell nanofibers^82^	340	100	34.9
In_2_O_3_@ZnO core–shell nanowires^74^	350	100	105
In_2_O_3_@ZnO core–shell nanowires^80^	300	200	18.8
In_2_O_3_@ZnO core–shell nanowires (this work)	400	100	245

Introducing ZnO as a shell material significantly boosts the performance of In_2_O_3_-based sensors. With a 10 nm ZnO coating, our core–shell sensor achieves a maximum response of ≈245 at 100 ppm of ethanol, comparable to that reported by Singh *et al.* for ZnO-coated (≈20 nm shell) In_2_O_3_ single-crystalline rod-like crystals (≈80 nm in diameter) grown by VLS, measured at 350 °C operating temperature.^74^ In contrast, Park *et al.* reported a response of 18.8 for a 27 nm ZnO coating at 200 ppm of ethanol,^80^ which is an order of magnitude lower than our result. Electrospun nanotubes composed of mixed indium and zinc precursors, as demonstrated by Huang *et al.*, reached a maximum response of ≈18.67 at 225 °C.^77^ Zhang *et al.* for the carambola-like Zn-doped In_2_O_3_ nanostructures demonstrated response ≈203 for the sensor operating at relatively low 220 °C and exposed to 50 ppm ethanol concentration.^81^ We attribute the exceptional performance of our sensor to the synergistic effect of the multi-stacked thin (∼24 nm) In_2_O_3_ NW architecture and the ZnO shell of an optimal thickness, allowing the detection of ethanol concentrations down to 1 ppm. Notably, the baseline resistances (*R*_0_) of all the tested samples remained in the low MOhm range or below (Table S6 and Fig. S12), comparable in magnitude to the mesoporous hydrothermally synthesized In_2_O_3_–ZnO system demonstrated by Jiang *et al.*,^83^ allowing for straightforward electrical measurements with standard laboratory equipment.

### 3.4. Stability of core–shell In_2_O_3_@ZnO nanowire meshes under operating conditions and sensor's cross-sensitivity to water vapor

Long-term stability tests (>12 hours) were performed for the sensor coated with a 10 nm ZnO shell, it was subjected to 80 cycles of exposure to 20 ppm ethanol (Fig. 7a). We note a slight baseline resistance drift throughout the operating time, which gradually stabilizes after approximately 20 cycles, converging to a steady *R*_0_/*R* value of ≈110 at 400 °C. These observations indicate that implementing a brief pre-operation aging step could further enhance signal stability. Compared to the initial measurements, the response matches quite well with the one shown in Fig. 6f, which emphasizes a notable stability even upon prolonged storage and cycling.

To evaluate the potential interference from ambient moisture, an additional humidifier supplying air with ≈40% RH was introduced, enabling assessment of sensor performance under ethanol exposure in moisture-enriched conditions, typical for ambient air in many indoor environments (Fig. 7b). In our experiment, the sensor was first subjected to 10 cycles of exposure to 20 ppm ethanol in dry air (≈3% RH), followed by an analogous series of cycles in humidified air. In the dry air, the sensor exhibited a stable and reproducible signal consistent with the behavior shown in Fig. 6f (black curve) along with data shown in Fig. 7a (Fig. 7b, black curve). In the humid air, a reduction in baseline resistance was observed, likely due to increased surface –OH residues density. A slight limitation of the experiment was that the interval between dry- and humid-air exposure was insufficient for the relative humidity to fully stabilize, with %RH in our test chamber reaching steady-state only after approximately four cycles. Extending the equilibration period to ≈1 h would eliminate this transient effect. Nevertheless, once stable humidity conditions were established, the sensor maintained consistent operation, demonstrating robust performance in the presence of water vapor.

## 4. Conclusions

We demonstrated a simple single-step synthesis of In_2_O_3_ nanowire meshes on Si/SiO_2_ substrates using block copolymer templating. The resulting porous structures functioned as efficient chemiresistive sensors, showing a response of ∼120 to 100 ppm ethanol vapor. Sensitivity was further enhanced by conformal ZnO coatings deposited *via* ALD, with an optimized ZnO layer thickness (∼10 nm) yielding a response of ∼245 under the same conditions and a maximum sensitivity of 2.28 ppm^−1^. These improvements are consistent with Debye length considerations and highlight the role of interface engineering in boosting sensor performance. Long-term operating stability measurements demonstrated that the core–shell configuration provides highly reproducible responses over repeated ethanol exposure cycles, confirming the robustness of the sensing architecture. Furthermore, ethanol detection tests conducted in humid air revealed only a minor reduction in response, indicating limited susceptibility to the humidity interference and emphasizing the practical applicability of the sensor in the indoor ambient atmosphere.

Overall, our findings underscore the importance of optimizing both morphology and processing conditions to control the crystallinity, porosity, and architecture of nanowire sensing meshes. For future integration of functional metal oxide nanomaterials into sensing devices, our approach offers a rapid and easily scalable route to core nanowire fabrication.^56^ By selecting appropriate BCP molecular weights and block compositions, one can precisely tune nanowire spacing and diameter, while the volumetric ratio provides an additional handle to tailor mesh morphology, enabling structures such as lamellar or gyroidal nanomeshes. Furthermore, performance can be optimized through the careful choice of shell composition and Debye-length-matched thickness, achieved *via* ALD. With further refinement, the presented fabrication strategy opens multiple avenues to address persistent limitations of MOS gas sensors, including selectivity toward specific VOCs, improved reproducibility, and enhanced long-term stability through reduction of the baseline drift. The solution-based templating route enables the incorporation of mixed transition-metal or other metal oxide domains, providing a direct means to tailor chemical specificity and supporting the development of integrated electronic-nose architectures on a single chip. Moreover, judicious control of core–shell structure composition and morphology, holds promise in achieving efficient sensor operation at lower temperatures. Continued investigation of the underlying physicochemical mechanisms governing charge transport and surface reactivity in these complex heterostructures will be essential for further optimization. Collectively, these directions substantially expand the technological relevance of the fabricated nanomesh heterostructures toward high-performance sensing platforms capable of reliable VOC detection at sub-ppm level.

## Author contributions

Conceptualization – PP and PWM; sample fabrication – PP, ZZ, JK; ALD deposition and photolithography patterning – MKr and PC; sensor electrical characterization: PP, ZZ, JK; XPS characterization, analysis and results description – MD, ML; SEM characterization – PP, ZZ; XRD characterization – PP, PWM; TEM characterization – MM; graphics preparation – PP, ZZ, JK; writing – original draft: PP; funding acquisition, validation, supervision – PWM. All authors contributed to the discussion, as well as review and editing of the final manuscript version.

## Conflicts of interest

There are no conflicts to declare.

## Supplementary Material

RA-016-D5RA07053H-s001

## Data Availability

The data that support the findings of this study are available from the corresponding author PWM upon reasonable request. Supplementary information (SI): SEM images presenting monolayer-thick morphologies of In_2_O_3_ NWs, cross-sectional view SEM morphologies of In_2_O_3_@ZnO core–shell NWs, a photo of a photothermal setup used for thermal gradient annealing experiment, PXRD patterns of uncoated indium oxide NWs before and after thermal annealing in an oxygen atmosphere, XPS spectra with detailed description of the calibration procedure, as well as gas sensor supplementary data for core–shell samples, including base resistance, raw resistance, response values, and calculated sensitivity for 6-layer thick In_2_O_3_ NWs series. See DOI: https://doi.org/10.1039/d5ra07053h.
