# Supplementary material for: High-sensitivity ethanol vapor detection using In2O3@ZnO core–shell nanomeshes fabricated via block copolymer templating
Source: RSC Adv. 2026 Jan 21;16(6):4694–707. doi: 10.1039/d5ra07053h (PMC12822549; doi:10.1039/d5ra07053h)
Supplement: RA-016-D5RA07053H-s001 [file RA-016-D5RA07053H-s001.pdf]

Supplementary Information (SI) for RSC Advances.
This journal is © The Royal Society of Chemistry 2026

Supplementary information

High-Sensitivity Ethanol Vapor Detection Using In_2O_3@ZnO Core-Shell Nanomeshes Fabricated via Block Copolymer Templating

Przemyslaw Pula[1], Zofia Z. Zawistowska[1], Julia Krol[1], Magdalena M. Majewska[1], Maciej Krajewski[2], Paulina Chyzy[2], Mariya V. Dobrotvorska[3], Maria Kaminska[2], Mikołaj Lewandowski[3], Pawel W. Majewski[1]**

*Email: lewandowski@uam.edu.pl; pmajewski@chem.uw.edu.pl

[1]Faculty of Chemistry, University of Warsaw, Warsaw, 02093, Poland

[2]Faculty of Physics, University of Warsaw, Warsaw, 02093, Poland

[3]NanoBioMedical Centre, Adam Mickiewicz University, Poznań, 61614, Poland

Contains:

Note 1 – Complementary SEM images of indium oxide NWs morphologies

Note 2 – High-temperature thermal gradient annealing setup

Note 3 – Supplementary PXRD patterns

Note 4 – XPS survey spectra and analysis details

Note 5 – Ethanol sensor supplementary data

Note 1 – Complementary SEM images showing indium oxide NWs morphologies

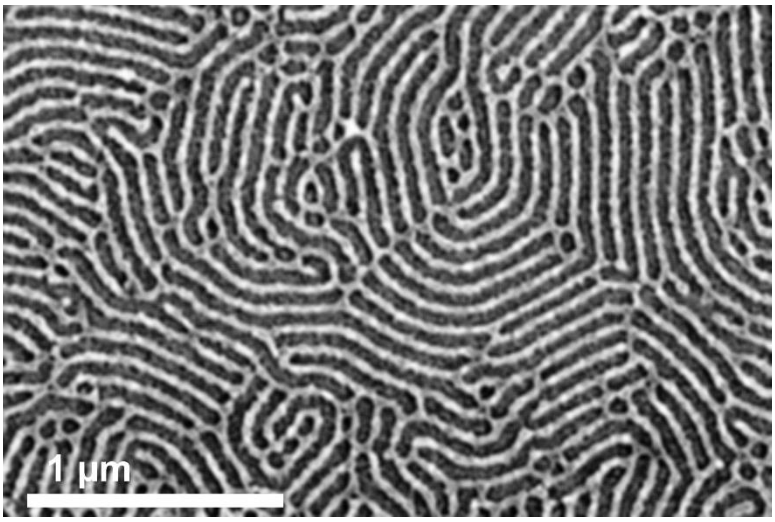

Fig. S1 SEM image of a single-layer thick In_2O_3 NWs replica of C259 PS-*b*-P2VP (In:VP 3:8) prepared from a 10% TMOT solution in toluene and spin-coated at 6000 rpm for 120 s. The film was subjected to an oxygen plasma ashing.

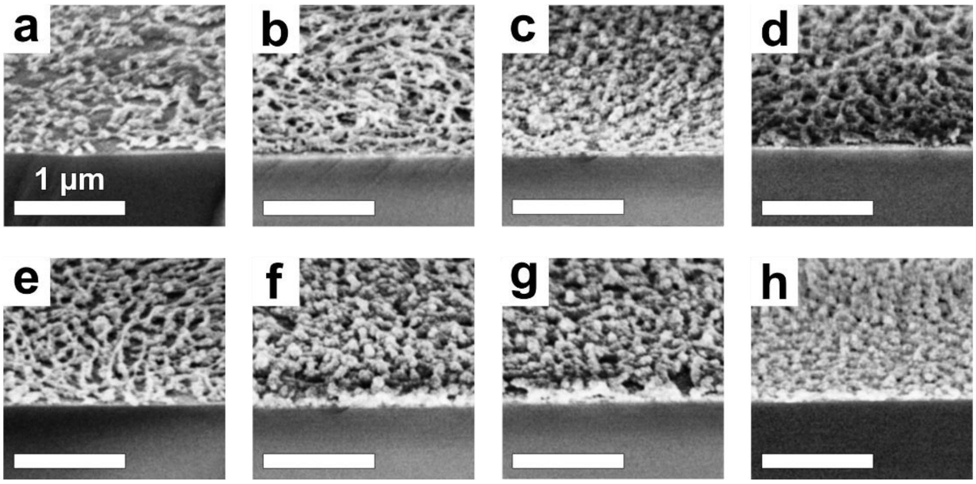

Fig. S2 Cross-sectional SEM images of oxygen-plasma ashed nanomeshes derived from for 4-layer- (a–d) and 6-layer-thick (e–h) C259 PS-*b*-P2VP films blended with indium (III) acetylacetonate at an In:VP stoichiometric ratio of 3:8. The images were acquired by tilting the carrier silicon wafers at 70° with respect to the optical axis. The panels show samples with varying ZnO coating thickness: no coating (a, e), 5 nm (b, f), 10 nm (c, g), and 20 nm (d, h).

Note 2 – High-temperature thermal gradient annealing setup

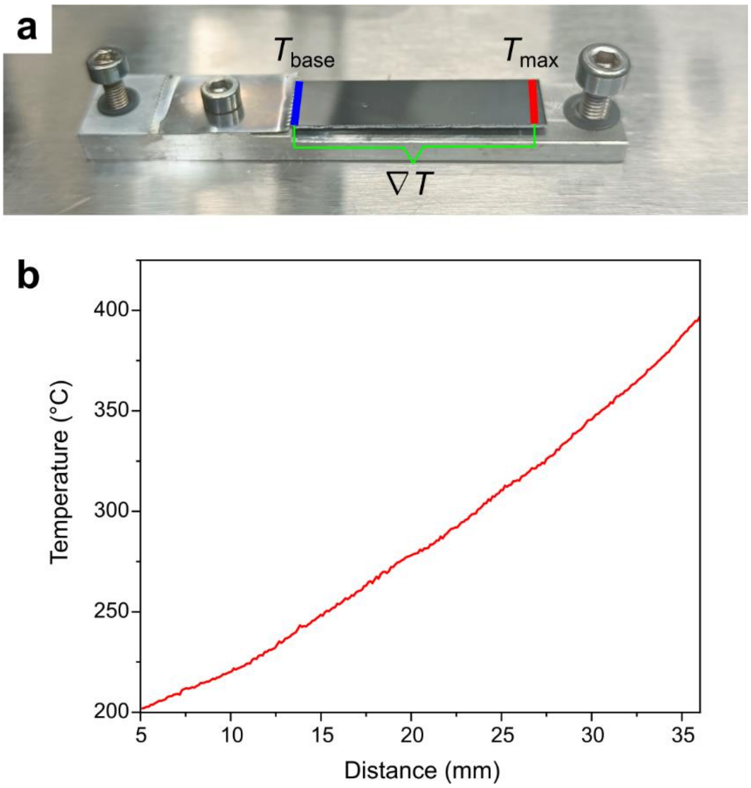

Fig. S3 (a) Photograph of the custom-made photothermal annealing setup ("diving board") enabling thin film annealing over a range of temperatures with the mounted silicon substrate. The blue line indicates the region corresponding to the base temperature, while the red line marks the distal end of the substrate, where the laser line is incident. The green bracket highlights the area over which the thermal gradient is established. (b) Temperature profile extracted from the Optris Xi400 thermal camera software, illustrating the thermal gradient shown in Fig. 3b at base temperature of 200 °C and 30 W laser power, established on the silicon substrate under ambient air conditions.

Note 3 – Supplementary PXRD patterns

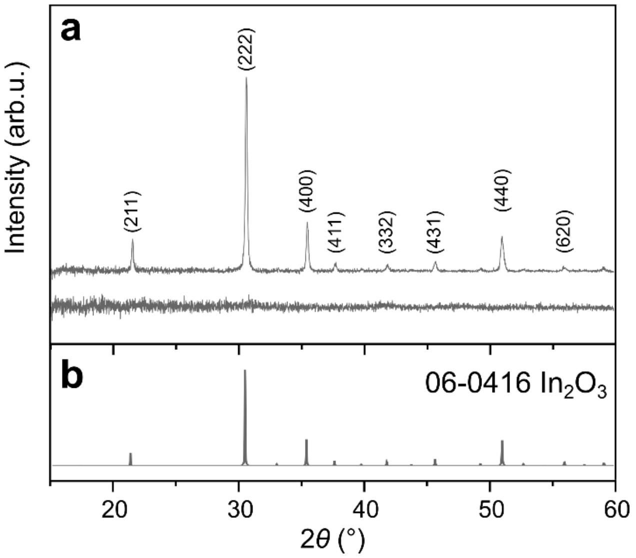

Fig. S4 (a) XRD pattern of In_2O_3 C259 replica cast on a silicon wafer, recorded before (bottom curve) and after annealing in O_2 at 400 °C for 5 minutes (upper curve). The identified *hkl* crystallographic planes are marked for the respective reflexes of In_2O_3. (b) A simulated diffraction pattern of In_2O_3, derived from the respective CIF entry, alongside the corresponding JCPDS card number.

Note 4 – XPS survey spectra and analysis details

Tab. S1 XPS-derived elemental composition of 4-layer thick In_2O_3 nanowire meshes with respective ZnO coatings.

Sample	O 1s	Au $4f_{7/2}$	C 1s	Si 2s	In $3d_{5/2}$	Cr $2p_{3/2}$	N 1s	Zn $2p_{3/2}$
**In_2O_3 NWs**	51.6%	12.0%	13.7%	13.7%	7.9%	1.1%	-	-
	532.6 eV (77%)<br>530.5 eV (23%)	84.0 eV	285.3 eV (88%)<br>288.0 eV (12%)	154.0 eV	445.1 eV	576.3 eV	-	-
**In_2O_3 NWs + 5 nm ZnO**	43.0%	-	25.7%	-	-	-	4.5%	26.8%
	531.7 eV (40%)<br>530.2 eV (60%)	-	285.3 eV (66%)<br>288.2 eV (34%)	-	-	-	399.5 eV	1021.4 eV
**In_2O_3 NWs + 2 nm ZnO**	43.9%	9.8%	20.1%	13.8%	6.1%	Overlap with Zn Auger	1.4%	4.9%
	532.6 eV (79%)<br>530.7 eV (21%)	84.0 eV	285.5 eV (70%)<br>288. 3eV (30%)	154.4 eV	445.1 eV		400.2 eV	1022.6 eV

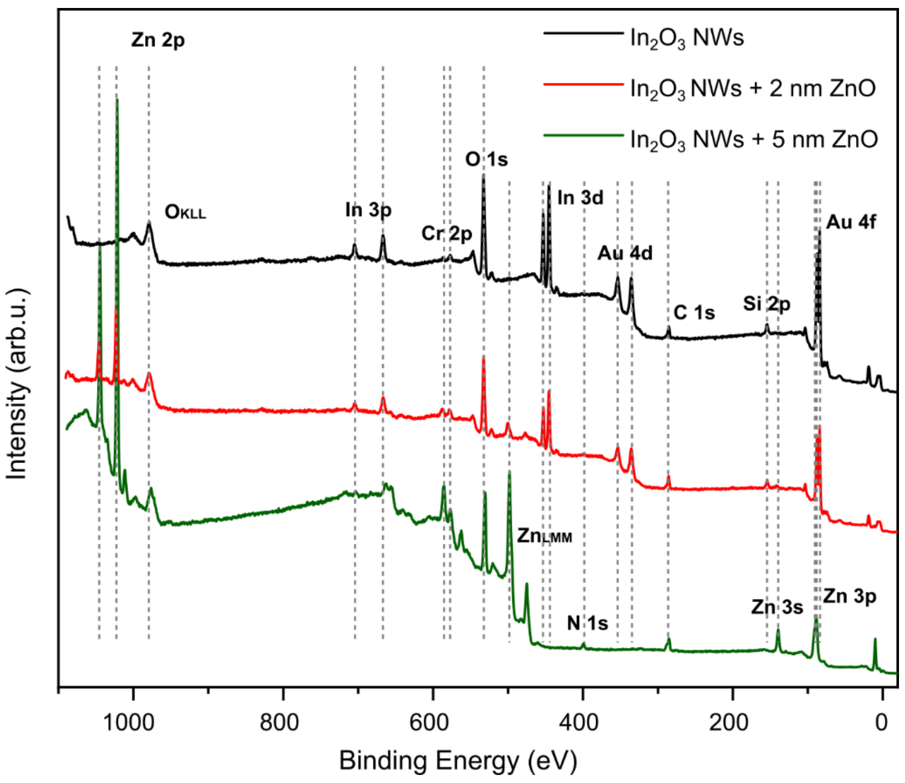

Fig. S5 XPS survey spectra of 4-layer thick In_2O_3 nanowire meshes (black curve), and In_2O_3 nanowires coated with 2 nm (red curve) and 5 nm (green curve) ZnO layers.

Detailed information on the chemical states of respective elements was obtained by fitting the recorded XPS lines and analyzing the positions of fitted components. The oxygen lines could be fitted with two components, one centered at around 532 eV, related to adsorbed oxygen, oxygen bonded to silicon, carbon or hydrogen – and one at 530 eV, which is characteristic of oxygen in metal oxides. The components contributing to the carbon C 1s line could be assigned to adventitious hydrocarbons (285 eV) and the C=O bonds (~288 eV). The position of N 1s (399–400.2 eV) was characteristic of adsorbed nitrogen. The Si 2s line was positioned at 154 eV, which indicated the presence of silicon dioxide SiO_2 (which is common for silicon wafers). The position of the Cr 2p line pointed to the presence of chromium oxide.

Note 5 – Ethanol sensor characteristics supplementary data

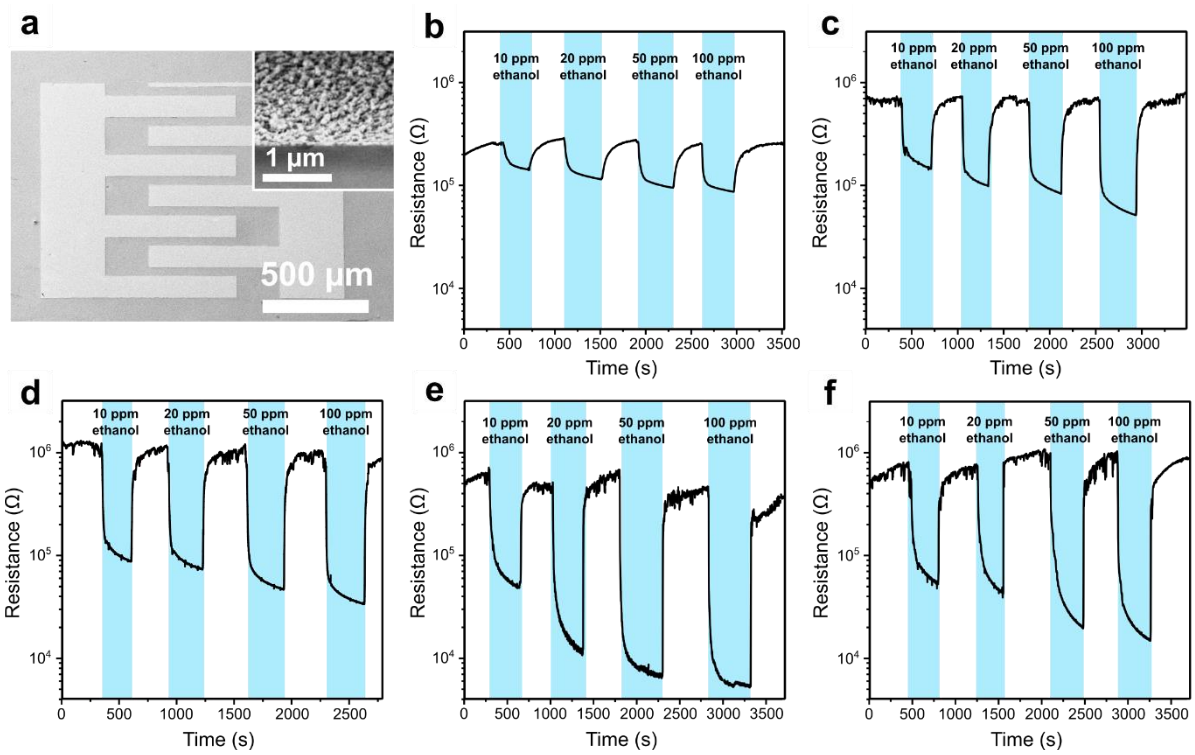

Fig. S6 (a) Top-view SEM image of a 4-layer thick In_2O_3@ZnO NW mesh with Au contact electrodes, fabricated from the C259 template using an In:VP ratio of 3:8. Inset shows the tilted-view SEM image of the sensory NW mesh. The NWs have a 10 nm-thick ZnO shell and were annealed in O_2 at 400 °C for 5 minutes. (b–f) Sensor raw resistance curves recorded for this ZnO-coated nanomesh at operating temperatures of (b) 250 °C, (c) 300 °C, (d) 350 °C, (e) 400 °C, and (f) 450 °C. Each curve shows the sensor's resistance change to sequential 5-minute exposure to 10, 20, 50, and 100 ppm of ethanol delivered in the dry air. Each exposure is followed by a 5-minute purge with pure air.

Tab. S2 Response values recorded after 5 min of ethanol exposure at various concentrations for the sensor shown in Fig. 6, measured at different operating temperatures. The linear fit equations were determined using the least-squares method.

**Temperature [°C] / Ethanol concentration [ppm]**	**10**	**20**	**50**	**100**	**Sensitivity**	**Linear fit equation**
**250**	1.7	2.2	2.6	2.9	0.012	y = 1.01199x + 1.80296
**300**	7.5	11.0	13.2	21.0	0.139	y = 0.13949x + 6.89796
**350**	12.5	15.0	23.5	33.0	0.229	y = 0.22857x + 10.71429

**400**	12.0	108.0	162.0	245.0	2.279	y = 2.27857x + 29.21429
**450**	29.5	62.0	153.5	202.5	1.903	y = 1.90255x + 26.2602

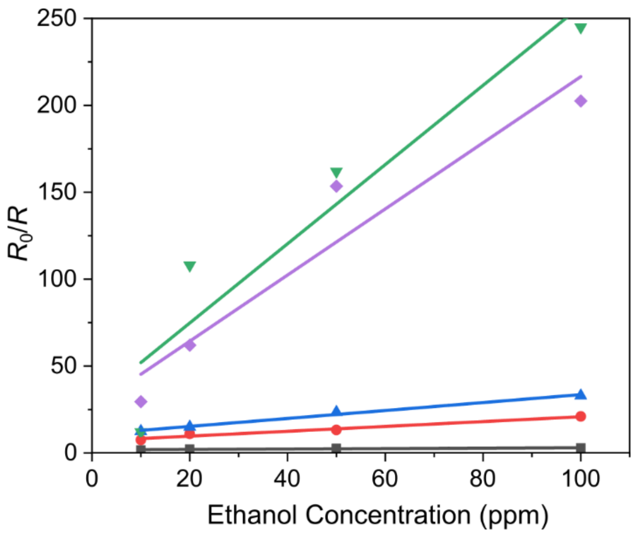

Fig. S7 Response values collected after 5 min of ethanol exposure for the sensors shown in Fig. 6 with the best-fit straight lines for sensitivity at operating temperatures of 250 °C (black), 300 °C (red), 350 °C (blue), 400 °C (green), and 450 °C (purple).

Tab. S3 Response values recorded after 5 min of ethanol exposure at various concentrations for sensors shown in Fig. 6f with varying with ZnO layer thickness. The linear fit equations were determined using the least-squares method.

Sample / Ethanol concentration (ppm)	10	20	50	100	Sensitivity	Linear fit equation
**Bare In_2O_3**	20	56	81	121	1.012	y = 1.0122x + 23.949
**In_2O_3@ZnO 5 nm**	12	32	50	100	0.925	y = 0.9245x + 6.898
**In_2O_3@ZnO 10 nm**	12	108	162	245	2.279	y = 2.2786x + 29.214
**In_2O_3@ZnO 20 nm**	30	41	52	65	0.359	y = 0.3592x + 30.837
**In_2O_3@ZnO 30 nm**	6	8	9	11	0.045	y = 0.0475x + 6.5

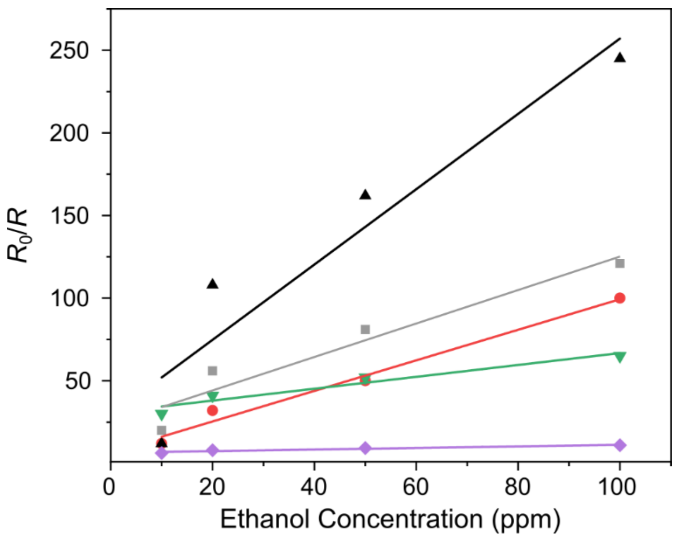

Fig. S8 Response values recorded after 5 min of ethanol exposure at various concentrations for 4-layer In_2O_3 sensors shown in Fig. 7 varying with ZnO layer thickness: 0 nm (gray), 5 nm (red), 10 nm (black), 20 nm (green), and 30 nm (purple) with the best-fit straight lines for sensitivity at 400 °C operating temperature.

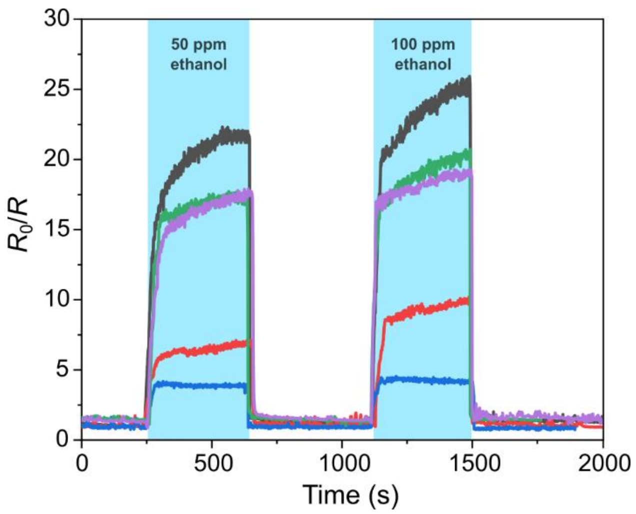

Fig. S9 Dynamic response characteristics obtained for ALD-derived flat ZnO layer of 5 nm (blue), 10 nm (red), 20 nm (black), 30 nm (green), and 50 nm (purple) thickness. All samples were annealed in O_2 at 400 °C for 5 minutes prior to testing. Sensing was conducted at 400 °C by exposing each sample to 50 ppm and 100 ppm of ethanol in 5-minute intervals.

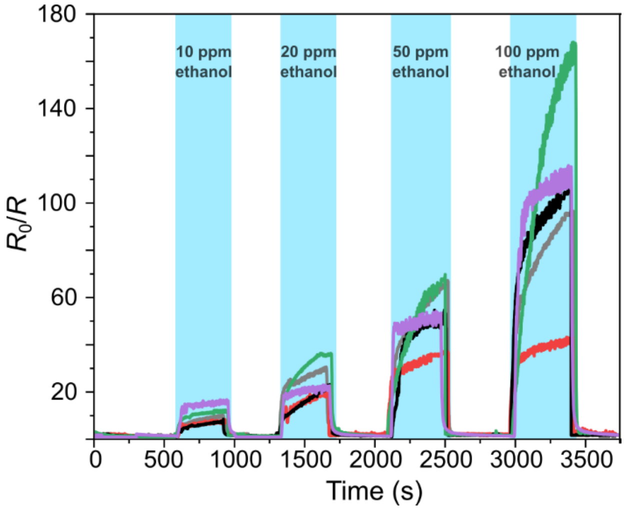

Fig. S10 Dynamic response characteristics obtained for 6-layer thick In_2O_3 nanowire meshes with varying ZnO shell thickness. The In_2O_3 nanowires, derived from the C259 template at an In:VP ratio of 3:8, were tested in their bare form (gray) and with 5 nm (red), 10 nm (black), 20 nm (green), or 30 nm (purple) ZnO coatings. All samples were annealed in O_2 at 400 °C for 5 minutes prior to testing. Sensing was conducted at 400 °C by exposing each sample to 10 ppm, 20 ppm, 50 ppm, and 100 ppm of ethanol in 5-minute intervals.

Tab. S4 Response values recorded after 5 min of ethanol exposure at various concentrations for the sensors shown in Fig. S9 varying with ZnO layer thickness. The linear fit equations were determined using the least-squares method.

**Sample / Ethanol concentration (ppm)**	**10**	**20**	**50**	**100**	**Sensitivity**	**Linear fit equation**
**Bare In_2O_3**	15	30	65	92	0.838	y = 0.8388x + 12.755
**In_2O_3@ZnO 5 nm**	9	22	33	44	0.351	y = 0.351x + 11.204
**In_2O_3@ZnO 10 nm**	7	22	55	108	1.106	y = 1.1061x - 1.7755
**In_2O_3@ZnO 20 nm**	13	39	62	180	1.792	y = 1.7918x - 7.1327
**In_2O_3@ZnO 30 nm**	17	22	52	115	1.110	y = 1.1102x + 1.5408

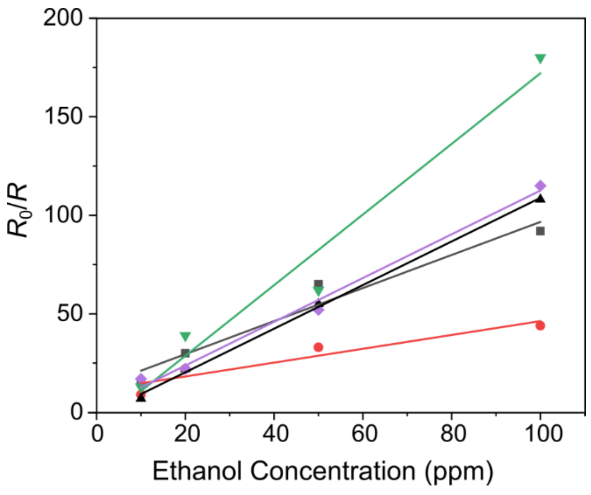

Fig. S11 Response values recorded after 5 min of ethanol exposure at various concentrations for the sensors shown in Fig. S9 with varying ZnO layer thickness: 0 nm (gray), 5 nm (red), 10 nm (black), 20 nm (green), and 30 nm (purple) with the best-fit straight lines for sensitivity at 400 °C operating temperature.

Tab. S5 Comparison of commercially available MOS ethanol and VOC sensors (as of 2025), summarizing key performance parameters including measurement range, and sensitivity (if reported). Manufacturer names have not been disclosed for legal reasons.

**Detected compound**	**Measurement range [ppm]**	**Sensitivity**
Ethanol	0.5–1000	R_0 (in air) / R_S (at 10 ppm ethanol) ≥ 2
Ethanol	1–50	R_0 (in air) / R_S (at 10 ppm ethanol) ≥ 5
Ethanol	0-500	R_0 (in air) / R_S (at 50 ppm ethanol) ≥ 3
Ethanol	25–500	R_0 (in air) / R_S (at125 ppm ethanol) ≥ 5
Ethanol	1–10	R_0 (in air) / R_S (at 10 ppm ethanol) > 2
Ethanol	10–1000	R_0 (in air) / R_S (at 50 ppm ethanol) ≥ 5
Ethanol	1–30	R_0 (in air) / R_S (at 10 ppm ethanol) $\approx$ 2–12

Tab. S6 Base electrical resistance in air (R_0) values measured for respective 4- and 6-layer-thick sensors before performing dynamic characteristics experiments. One should note that the base resistance values are in the order of magnitude of several MOhm.

**Sample**	R_0 **[MΩ]**
**In_2O_3 NWs 4x (400°C)**	4.5
**In_2O_3@ZnO 4x 5 nm (400°C)**	1.5
**In_2O_3@ZnO 4x 10 nm (250°C)**	0.25
**In_2O_3@ZnO 4x 10 nm (300°C)**	1.1
**In_2O_3@ZnO 4x 10 nm (350°C)**	1.1
**In_2O_3@ZnO 4x 10 nm (400°C)**	1.3
**In_2O_3@ZnO 4x 10 nm (450°C)**	0.78
**In_2O_3@ZnO 4x 20 nm (400°C)**	15
**In_2O_3@ZnO 4x 30 nm (400°C)**	4.0
**In_2O_3 NWs 6x (400°C)**	0.23
**In_2O_3@ZnO 6x 5 nm (400°C)**	0.07
**In_2O_3@ZnO 6x 10 nm (400°C)**	0.07
**In_2O_3@ZnO 6x 20 nm (400°C)**	4.4
**In_2O_3@ZnO 6x 30 nm (400°C)**	4.0

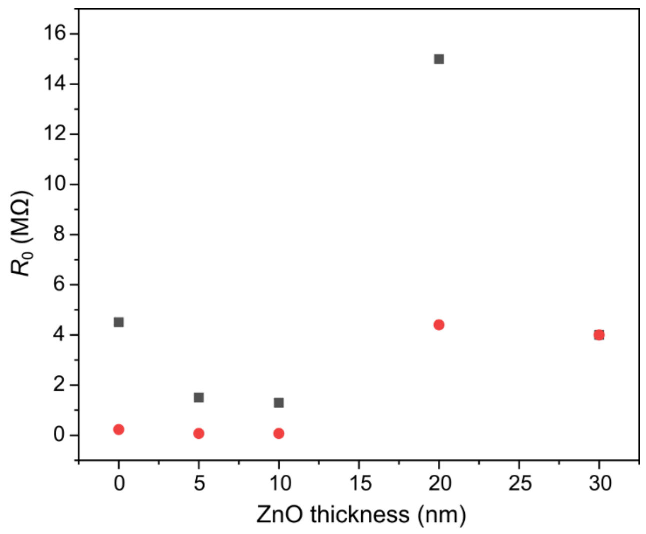

Fig. S12 Base electrical resistance in air (R_0) values plotted for respective 4- (red dots) and 6-layer-thick (black dots) sensors with respect to ZnO coating thickness before performing dynamic characteristics experiments. Points for 30-nm ZnO thickness overlap.
